# Progress of Surgery

**Published:** 1903-04-11

**Authors:** 


### Progress of Surgery.

(*Concluded from page* 10.)

**The Treatment of Gonorrhœa.**—Wolbarst[5] has come to the following conclusions with regard to the treatment of gonorrhœa with protargol. The earlier the treatment is begun the greater is the probability that implication of the posterior urethra will be avoided. Posterior urethritis is more likely to result if the meatus is narrow and early meatotomy is advisable in some cases. The irrigation treatment for posterior urethritis is rational, effective, and attended, when properly carried out, with a minimum of pain and danger. For the irrigation, potassium permanganate, normal salt solution, silver nitrate and copper sulphate have given equally good results. The heat of the fluid and the mechanical flushing are probably the most effective forces leading to the favourable results of the treatment. For anterior urethritis protargol in half to one per cent. solution is most valuable. It is neither irritant nor painful, and can be used from the beginning. The gonococci disappear after two to 16 days, but are apt to reappear if the protargol is stopped before the end of the third week. The average duration of treatment is six weeks. The profuse purulent discharge usually disappears in from seven to ten days and then is replaced by a muco-purulent or watery discharge. The latter disappears in two to four weeks with astringent injections. Disadvantages of the treatment are that the early cessation of the discharge induces patients to stop treatment before they are cured, and minimises the importance of the disease in their eyes. Posterior involvement may arise if the solution is too strong. G. K. Swinburne[6] advocates the use of a new silver preparation called argyrol. It contains nearly 30 per cent. of silver, whereas argonin contains 4 per cent. and protargol 8 per cent. The writer has used argyrol extensively. For irrigation the strength of the solution employed was from 1 in 2,000 up to 1 in 500, and for injection afterwards the strength was 1 to 5 per cent. The drug causes no pain. It has decided gonococcidal powers, a decided effect in reducing the inflammation, and can be used safely in almost any strength and at any stage of the disease. The author believes it to be one of the most valuable remedies for gonorrhœa given to the profession in recent years. C. Leedham-Green[7] has considered the treatment of chronic gonorrhœa. Two chief varieties of the disease are described, viz., (1) the sub-acute, in which there are localised areas of infiltration, and in addition a general catarrhal inflammation of the mucous membrane, with mucous as well as threads in the urine; (2) the inveterate or circumscribed, in which the trouble is confined to localised portions of the urethra. These may affect the mucous membrane only or the submucous tissue also. In the sub-acute variety the general catarrh must first be treated, and this is best carried out by irrigations with mild astringents, for the posterior urethra is generally involved. Simple nitrate of silver solution is preferable to the organic silver preparations, and should be used in a strength of from 1 in 10,000 to 1 in 500. Sulphate of zinc, 1 in 1,000 to 1 in 500, and permanganate of potash 1 in 10,000 to 1 in 2,000 are also useful. The irrigation is performed once a day until the urine is free from mucous. For the circumscribed disease, if confined to the mucous membrane, powerful astringents must be applied to the affected spot and to it only. This is best done through the endoscope. The solution used may be one of silver nitrate or copper sulphate in a strength of 1 per cent. to 10 per cent., and it is applied every two or three days. In the intervals the irrigations as described above may be carried out. Drugs may be applied in lanoline ointment to secure longer action, by means of an ointment introducer. A useful formula is nitrate of silver 1 to 5, lanoline 50, vaseline 50. If the disease has affected the deeper tissues mechanical dilatation will be required to induce absorption, and for this purpose the dilators of Oberländer and Kollman should be used.

**Catheterisation of the Ureters.**—Bransford Lewis, of St. Louis,[8] has contributed a paper on this subject, discussing the uses of the procedure and describing a catheterising cystoscope he has devised. The ureters may be catheterised for diagnosis or treatment. In diagnosis for the following purposes :—(1) To locate the origin of pus, blood, micro-organisms, and desquamated epithelium coming from the bladder, or the right or left kidney or peri-renal space ; (2) to determine the presence of two kidneys or, if one is present only, then which one ; (3) to determine the number of ureters present ; (4) to recognise and locate obstructions in the ureters from stricture, stone, adjacent tumours, kinks, or valves ; (5) to determine the relative functional activity of each kidney as indicated by the excreted urine from each ; (6) to determine the size and capacity of each kidney pelvis with respect to hydronephrosis, pyonephrosis, or total obliteration of the kidney-secreting tissue ; and (7) to estimate the relative amount of disease in each kidney, and whether it will be safe to remove one. In treatment the procedure may be used (1) to enlarge strictures or narrowings in the ureters, and possibly thus improve such conditions as pyelitis or allow calculi to pass ; (2) to irrigate the ureters or pelves ; (3) to use the ureter with the catheter *in situ* as a guide in certain abdominal operations ; and (4) by prolonged catheterisation of a ureter to assist in the cure of ureteral fistula. The writer quotes cases illustrating some of these uses. The most striking feature of the Bransford-Lewis cystoscope is that it is used with warm air in the bladder instead of watery solutions. The lamp in the beak is a cold one, and will not burn the bladder wall. With the most recently constructed instrument the two ureters can be very easily catheterised simultaneously. In estimating the relative functional capacity of each kidney it is essential to do this, as shown by Casper.

[5] Vide Abst. Practitioner, Oct. 1902, p. 489. [6] Med. Rec., Oct. 11, 1902, p. 574. [7] The Midland Med. Jour., Feb. 1903, p. 18. [8] Annals of Surg., Jan. 1903, p. 22.

